# A first estimate of the structure and density of the populations of pet cats and dogs across Great Britain

**DOI:** 10.1371/journal.pone.0174709

**Published:** 2017-04-12

**Authors:** James Aegerter, David Fouracre, Graham C. Smith

**Affiliations:** National Wildlife Management Centre, Animal and Plant Health Agency, Sand Hutton, York, United Kingdom; Universidade do Porto Instituto de Biologia Molecular e Celular, PORTUGAL

## Abstract

Policy development, implementation, and effective contingency response rely on a strong evidence base to ensure success and cost-effectiveness. Where this includes preventing the establishment or spread of zoonotic or veterinary diseases infecting companion cats and dogs, descriptions of the structure and density of the populations of these pets are useful. Similarly, such descriptions may help in supporting diverse fields of study such as; evidence-based veterinary practice, veterinary epidemiology, public health and ecology. As well as maps of where pets are, estimates of how many may rarely, or never, be seen by veterinarians and might not be appropriately managed in the event of a disease outbreak are also important. Unfortunately both sources of evidence are absent from the scientific and regulatory literatures. We make this first estimate of the structure and density of pet populations by using the most recent national population estimates of cats and dogs across Great Britain and subdividing these spatially, and categorically across ownership classes. For the spatial model we used the location and size of veterinary practises across GB to predict the local density of pets, using client travel time to define catchments around practises, and combined this with residential address data to estimate the rate of ownership. For the estimates of pets which may provoke problems in managing a veterinary or zoonotic disease we reviewed the literature and defined a comprehensive suite of ownership classes for cats and dogs, collated estimates of the sub-populations for each ownership class as well as their rates of interaction and produced a coherent scaled description of the structure of the national population. The predicted density of pets varied substantially, with the lowest densities in rural areas, and the highest in the centres of large cities where each species could exceed 2500 animals.km^-2^. Conversely, the number of pets per household showed the opposite relationship. Both qualitative and quantitative validation support key assumptions in the model structure and suggest the model is useful at predicting the populations of cats at geographical scales important for decision-making, although it also indicates where further research may improve model performance. In the event of an animal health crisis, it appears that almost all dogs could be brought under control rapidly. For cats, a substantial and unknown number might never be bought under control and would be less likely to receive veterinary support to facilitate surveillance and disease management; we estimate this to be at least 1.5 million cats. In addition, the lack of spare capacity to care for unowned cats in welfare organisations suggests that any increase in their rate of acquisition of cats, or any decrease in the rate of re-homing might provoke problems during a period of crisis.

## Introduction

In Great Britain (GB) pet cats and dogs are important to the economy, society, the environment and therefore to government. With almost half of households (46%) owning either at least one cat or dog [1] (hereafter collectively pets), the crude product of the approximate population size of each species (10 million animals [1]), and the current cost of dog ownership of approximately £1000 p.a., or £500 p.a. for cats (no robust estimates of these costs exist for GB though various contemporaneous commercial estimates around these figures are produced by market research companies using undescribed methodologies), suggest that pet ownership generates approximately £15 billion p.a. across the GB economy. Although the ownership of pets in GB is thought to bring physical and psychological benefits to their owners [2–5] and thus to society as a whole (given the frequency ownership), it is also associated with social problems such as toxocariasis [6–9], toxoplasmosis [6,10–12], asthma or other allergies, e.g. [13,14], as well as problems for the environment (reviewed for GB in [15–17] and see [18,19]). Thus even without the specific consideration of pet diseases of policy concern, both cats and dogs are of general interest to many aspects of government.

Government has a direct role in managing zoonotic diseases of policy concern (e.g. rabies) and may also have a role in the management of veterinary diseases of social concern. Sound policy development and effective contingency response rely on strong evidence to ensure success and cost-effectiveness. Accurate fine-scale maps of the density of pets would be helpful in assessing or predicting alternative policy scenarios, as well as the delivery of effective contingency response to a pet disease of policy concern. For example, to produce useful estimates of the number of pets within statutory control or surveillance zones, or estimates of the number of animals which rarely, or never see veterinarians, or might not be appropriately managed in the event of a disease outbreak. Unfortunately, such evidence for GB does not exist as the mandatory registration of dogs was only required from April 2016 and cat ownership requires no official notification at all.

A number of studies associating pet ownership with various social or human demographic (hereafter demographic) descriptions of their owners have been made for GB [1,20–22], though none of these have explicitly described the spatial variation in the density of pets. Downes *et al*. (2011) used demographic variables to predict the relative distribution of cats and dogs across the island of Ireland based entirely on descriptions of households. However their maps were unvalidated and a transposition of their approach to GB would be inappropriate. In order to provide this first quantitative description for the density of pets we have used an alternative approach and identified one of the few authoritative sources of spatially explicit data relating to their distribution; the activity and density of the veterinary services supporting them. Other potential direct descriptors of pet density, such as the sales of food or accessories as well as insurance services were not available (as all are commercially sensitive) and the few available estimates were of unknown quality and at inappropriately large spatial scales (e.g. advertising regions). In addition, predictions based on measures associated with economically discretionary consumables are all are subject to multiple and interacting un-estimable and ephemeral biases and were considered unsuitable.

We produce the first estimate of the density of pets at a fine spatial scale in order to provide an evidence base for decision-makers and other research workers, as well as providing a framework to support subsequent improved estimates. In addition, we attempt to produce the first strategic overview of pet cat and dog populations across GB, to permit other studies to be set in context as well as to identify important gaps in current knowledge. To do this we collate a number of recent estimates of the sizes of specific pet sub-populations (describing a range of ownership classes) or their rates of interaction, and describe the structure and dynamics of the populations of pets in GB. We use this to suggest the current population size of feral cats and identify potential issues with the management of a number of cat sub-populations in the event of an outbreak of a disease of policy concern.

## Methods

The most recent robust (scientific) estimate of the national populations of pets, 11,599,824 dogs (95% CI: 10,708,070–12,491,578) and 10,114,764 cats (95% CI: 9,138,603–11,090,924) [1] is subdivided in order to produce both a spatial description of pet ownership, and estimate key sub-population sizes (defined by the behaviours of their owners) in order to produce a coherent scaled description of the structure of the national populations of pets. Here we provide a brief overview of the rationale and approaches we used for both spatial and structural models. Detailed descriptions of data sources and software processes as well as aspects of the spatial modelling methodology are given in S1 Text, which can be read as an independent and complete description of our approach. The review of the recent literature describing component sub-populations of pets, which informs our description of the structure of the national pet populations, is given in S2 Text.

### Map

As veterinary practises in GB operate in a free-market, their distribution and activity should approximate the density of their clients; thus practise location and size were used to model the density of owned pets. Isochrones describing client travel time (i.e. accessibility) were used to define exclusive catchments around practises, and these were combined with maps of residential addresses to inform a spatial description of pet ownership and density. Locations of veterinary activity were obtained from the authoritative register of prescribing and dispensing practises maintained by the Royal College of Veterinary Surgeons (RCVS) along with a count of the number of veterinarians registered at each. As practises differ substantially in activity (i.e. the number of vets practicing) it cannot be assumed that every catchment serves an equal number of pets and in the absence of informative data a simple asymptotic relationship was used to represent the assumption that larger practises were more likely to employ part-time staff or specialists which would result in a proportional reduction in their annual rate of consultations for cats or dogs. Thus we express the activity of practises as full time equivalents (FTEs). The density of pets was calculated by dividing the national population across all catchments, weighted by the FTE (to produce an estimate of the pet population within each catchment), and then further subdivided into a fine-scale map of residential areas (postcode unit). These fine-scale estimates were geographically smoothed to relax the strict assumption that owners living close to catchment boundaries will only use their most accessible practise. The number of pets and the number of households was then aggregated into larger scale maps, e.g. quantitative validation required a 1km raster of cat density (aligned to the British National Grid), whilst the variation in the number of pets.household^-1^ across GB was represented at the scale of the postcode district.

#### Map validation

The only two recent and spatially explicit empirical estimates of cat density in GB were used to perform a quantitative validation of the map [19,24]. No similar data were available for dogs. Both studies undertook surveys across very small areas (e.g. a street) to describe the number of owned cats and extrapolated densities to 1 km^2^. These data were mapped and the empirical estimates compared with those modelled here using the intraclass-correlation coefficient (ICC), which varies between 0 (no correlation) and 1 (perfect correlation between observations) and produces two useful metrics; consistency and agreement. These respectively describe the similarity of density estimates at a location around an unknown mean, and a more generalised comparison of the trends shown by model and empirical data.

Whilst our model is based on an estimate of pet population size based on data collected in 2011 [1], we are required to use validation data collected in 2004–2005 [19] and 2008–2010 [24]. An alternative estimate of pet population size was available based on data collected in 2007 [20] which might have been used to correct some or all of our empirical validation data into a common time-frame. However we chose not to do this for two reasons. Firstly, both estimates of the national population are very similar and were considered statistically indistinguishable. Consequently, with only two indistinguishable estimates it is impossible to assume a temporal trend in national population estimate and extrapolate this to permit a correction for every date applicable in this study (range 2004 to 2013). Secondly, even if a proportional correction based on the mean estimate could be applied, it would be so small (≈2%) that no change in the ICC and quality of the quantitative validation is expected.

### Structures of pet populations

The national populations of cats and dogs were sub-divided into sub-populations described by their ownership and lifestyle. A matrix of ownership classes were defined following a review of the literature (S2 Text) and considered factors of importance when managing a disease of policy concern, principally; the degree of control over the pet, and its access to veterinary care. These then permit us to produce estimates of those animals that may not be well managed during a disease crisis (e.g. those animals which may either present late to a veterinarian, not present at all, or may not be under control). Our goal is to attempt to capture and then describe all key sub-populations as well as their interactions to permit a quantitative overview of this economically important activity. Estimates of the key sub-populations and their rates of interaction were then scaled to produce the first coherent contemporaneous description of the structure of the national cat and dog population in GB.

## Results

### Map

**Fig 1:**
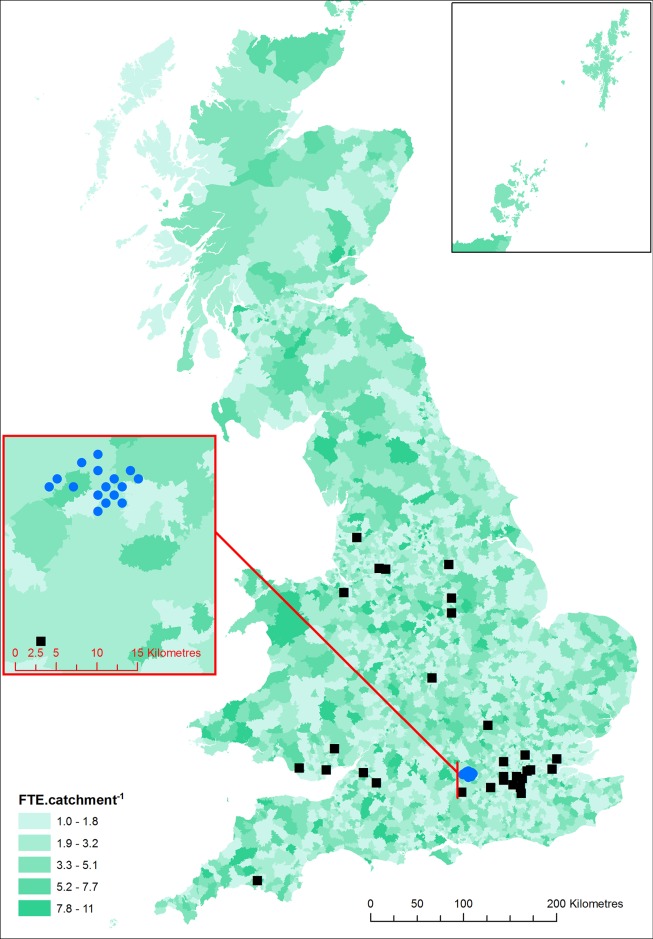
Catchment areas around mapped veterinary practises and the locations of validation data. Catchment areas (green with grey boundary); Data from Sims *et al*. (2008) black filled squares; Data from Thomas *et al*. (2012) blue filled circles.

The addresses of qualifying practises were acquired from the RCVS in late 2013, and additional data including the measure of practise size in early 2015. The map of all 3918 catchments across GB (Fig 1) represented 3992 practises. Catchments range in size from the geographically small (15 ha representing 452 homes in Tewkesbury, Gloucestershire), to 5,134 km^2^ (representing 10,330 homes) across the western highlands of Scotland. Similarly, some practises located in industrial estates apparently served only a few homes (e.g. 35 in Washington, Tyne and Wear) whilst others provide services for over 43,000 (around Handsworth, Birmingham). A number of catchments included more than one practise, either because multiple independent businesses were located very close together (within a single postcode unit), or on occasion the modelling technique produced catchment artefacts which were addressed by merging neighbouring catchments. The mapped practises represented 15,905 registrations of veterinary surgeons, though veterinarians may be registered at more than one location depending upon the practise business model; varying from sole operators (either independent or as part of a franchise), to large businesses employing over 30 vets at a single site or across a network of practices. We have assumed that this represents 11,799 FTE surgeons available to treat pets.

As the estimate of the national population of pets is expressed within an error structure (i.e. 95% confidence intervals [1]), predictions derived from its use must reflect this. Fortunately, the symmetrical variation in the national estimate of pet population sizes, as well as its simple application in our model, permits us to apply the confidence intervals proportionately to our predictions. For brevity, in this study we restrict our descriptions and discussion of pet density and the rate of ownership to the mean (most likely) estimate and its predictions: However alternative minimum and maximum metrics (e.g. densities or rates of ownership) can be simply derived by applying a ±9.7% or ±7.7% adjustment to the predicted mean metric for cats and dogs respectively. Thus although the most active catchment is most likely to host 11,558 dogs, this might represent as few as 10,668 or as many as 12,448. Similarly our calculations assume 1FTE ≈ 983±76 dogs and 857±83 cats.

**Fig 2:**
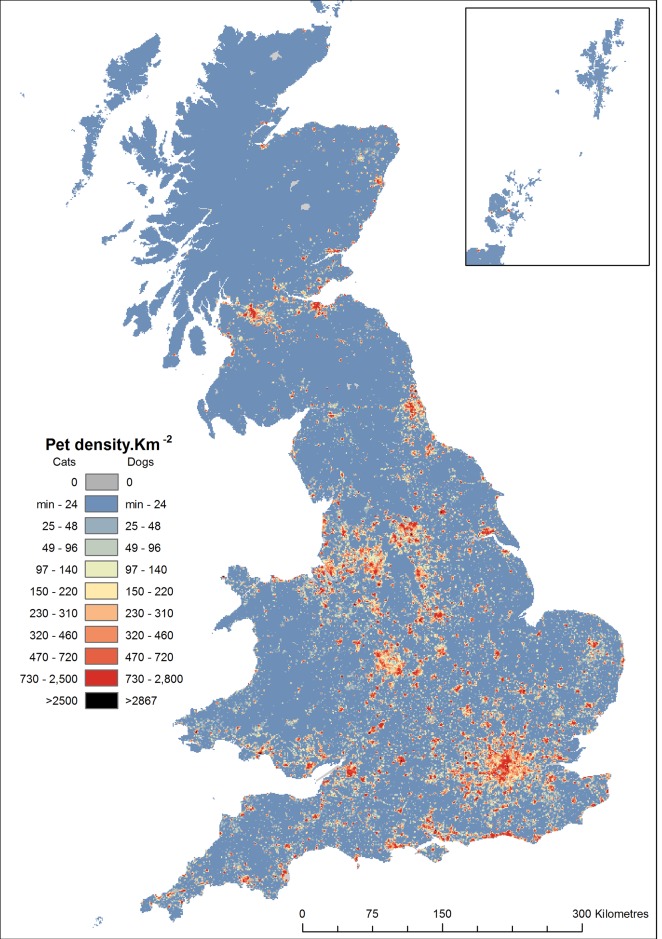
Density of pet cats and dogs across GB. Density predicted from mean population estimates; predictions of density derived from minimum and maximum population estimates can be produced by adjusting values shown here by ±9.7% for cats and ±7.7% for dogs.

**Fig 3:**
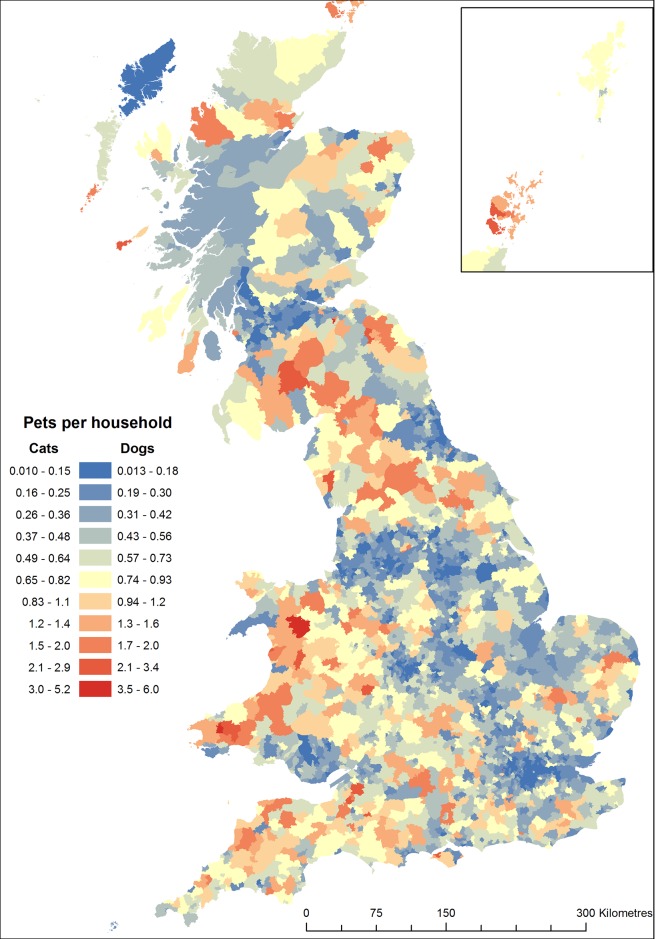
Spatial variation in the number of pet cats and dogs per household. Rate of ownership predicted from mean population estimates; predictions of ownership derived from minimum and maximum population estimates can be produced by adjusting values shown here by ±9.7% for cats and ±7.7% for dogs.

Densities of pets were highest in major metropolitan areas (Fig 2), whilst the number of pets.household^-1^ was generally highest in rural areas (Fig 3). The former was driven by the substantially higher density of homes in urban areas, which in turn has dictated the largely urban/suburban distribution of veterinary practises and the observed variation in the size and activity of their catchments (Fig 1). Conversely, the relative density of veterinary services in rural areas, combined with a low or very low density of homes produces the increase in the rate of ownership. For the first time we describe the spatial variation in the density of pets in diverse rural and urban settings across the country. For example we contrast the differences in pet populations in rural southern England, as compared to areas of rural northern England, northern Wales or large areas of Scotland; as well as the unexpected range of pet density and rates of ownership in the urban centres of British cities (Fig 1). We identified a few small areas on mainland GB where no pets were predicted; the most obvious of these were centred on ski-resorts, which host geographically large post-code units without residential addresses.

**Fig 4:**
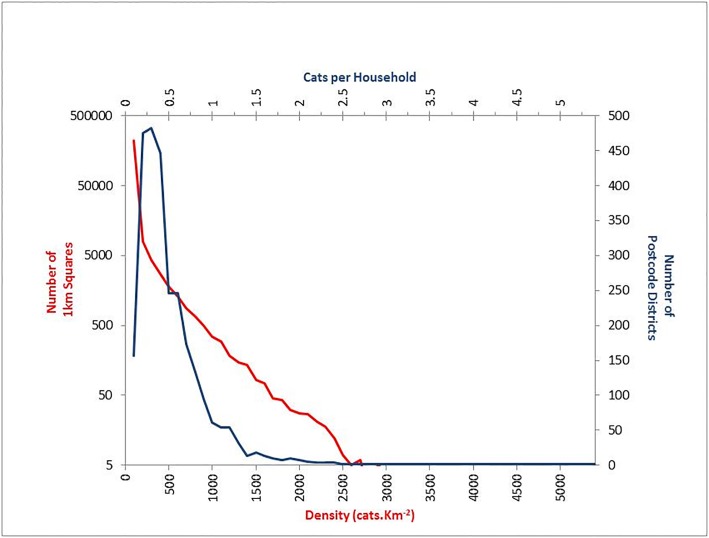
Frequency of modelled density and ownership measures across GB.

The predicted density of pets aggregated to a 1 km raster (Fig 2) varied from a minimum 0.002 pets.km^-2^ to a maximum of 5363 cats.km^-2^ and 5869 dogs.km^-2^ (excluding grid cells that were not entirely occupied by residential post codes). The predicted rates of ownership, aggregated to postcode districts varied from a minimum 0.01 pets.household^-1^ to a maximum 5.2 cats.household^-1^ or 5.9 dogs.household^-1^ (Fig 3). Most grid cells represent densities considered to be realistic (Fig 4), arbitrarily considered to < 2,500 cats.km^-2^ with only 0.001% exceeding this threshold. Similarly, most rates of ownership were predicted to be at plausible rates (e.g. <0.5 pets.household^-1^) with only a small proportion of postcode districts exceeding 1 pets.household^-1^ (Fig 4). The few discrete locations predicting apparently un-realistic densities of pets are generally independent of those indicating un-realistic rates of ownership though both are often associated with rural areas.

#### Validation

**Fig 5:**
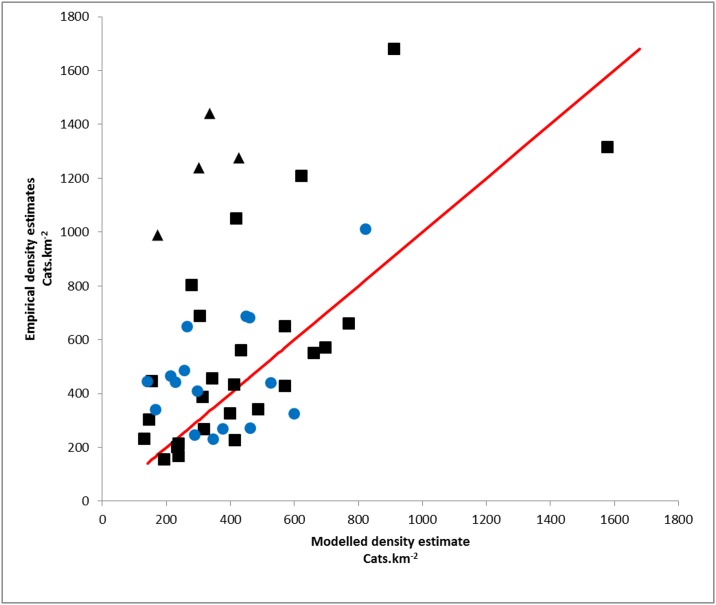
Comparison of empirical and modelled densities of cats. Data from Sims *et al*. (2008) as solid black markers; Data from Thomas *et al*. (2012) as solid blue markers. Four notable data points (see Discussion) shown as diamonds. Predicted densities shown as a red line for comparison.

Empirical densities of owned cats could be compared with our modelling approach at 46 locations (Fig 1) derived from two independent studies [19,24]. Modelled estimates at individual locations (1 km grid squares) loosely agreed with the empirical estimates, with areas predicted to host more cats generally doing so (ICC agreement of 0.35 and 0.43 for each study respectively: Fig 5). Our modelled estimates fell well within the general variation of both empirical studies (ICC consistency of 0.35 and 0.48 respectively). Additionally, we note the general distribution of empirical estimates both above and below modelled densities suggested an absence of bias in the predictions at these small scales (1 km^2^), and in turn that our model may simulate mean or median densities at larger spatial scales well (as these represent collections of unbiased independent small scale observations contributing towards an unbiased mean). Four densities reported by Sims *et al*. (2008) appear substantially higher than those estimated by the model (Fig 5); three of these are in areas of high or very-high density housing in urban London and one in a similar context in the centre of Cardiff.

### Structure of the national population of dogs in GB

**Fig 6:**
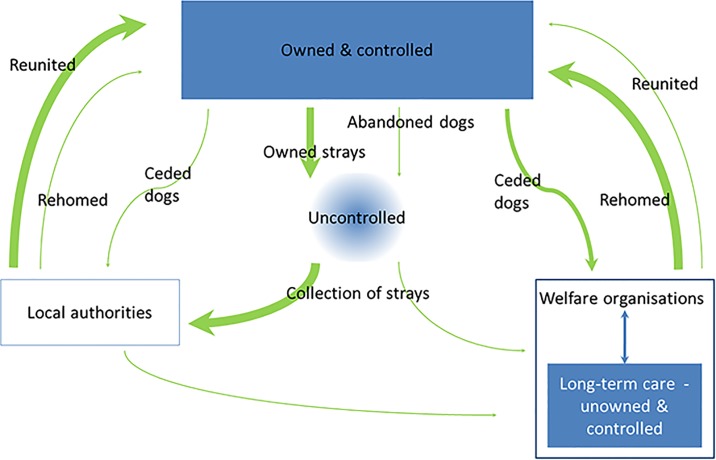
The structure of the British dog population: Sub-populations and their interactions.

Ownership classes for British dogs appear comparatively simple, exclusive and well described (Fig 6); being almost completely restricted to two standing sub-populations; owned & controlled, or unowned & controlled (S2 Text). Most owned dogs will be registered with a veterinary practise (77%, [25]) and most unowned dogs are housed by welfare organisations and will have timely veterinary support. Unlike many other countries there appears to be no consistent or measurable population of free-living unowned dogs, either as supported strays (i.e. receiving food, even irregularly, or occasional veterinary support), or as completely self-sustaining feral packs. The owned population appears largely to be kept under control, with only a very small proportion straying (Table 1 and S2 Text); of these an even smaller proportion represent abandoned pets (as opposed to those voluntarily ceded to local authorities or welfare organisations; S2 Text). In general strays are reported and promptly collected, either by local authorities or welfare organisations, and it is not thought that these dogs are uncontrolled for long; they represent a very small and transitory sub-population (Table 1). Most strays are recycled back into the owned sub-population and are either reunited with their owners or rehomed (Table 1) or remain in the care of welfare organisations.

**Table 1:** Quantitative estimates of dog populations and their interactions.

		Source				Working estimate
		Clark *et al*. (2012)	Stavisky *et al*. (2012)	Murray *et al*. (2015)	Anon. (2014)	
		Survey of welfare organisations (2009)	Survey of welfare organisations (2010)	Survey of households (2011)	Survey of Local Authorities (annual)	Mean estimate [mean % of owned dogs]
Estimated population of owned dogs				11,599,824		**11,599,824**
Estimate of dogs entering care.year^-1^		129,743	89,571			**109,657 [0.0095%]**
Stray dogs*					97,000 (2008) 126,176 (2011) 110,000 (2014)	**113,000 [0.01%]**
% of welfare dogs recycled into the owned population	reunited	12.5%	7.1%			**9.8%**
	rehomed	61.3%	75%			**68.2%**
Standing population of dogs in welfare organisations*		11,207 (368 of 1364 responses)	10,630 (259 of 536 responses)			**21,837 [0.0019%]**

* For illustration we have included three values for the period 2008–2014; the lowest, the highest (contemporaneous with the key population estimate for owned dogs), and the most recent available.

When considering the evidence required by decision-makers planning or undertaking a response to an outbreak of disease of policy concern in dogs, a number of key gaps in evidence have been identified. These include a refinement of the estimate of the proportion of owned dogs receiving little or no veterinary support (especially its spatial variation), a description of the mean period for which a dog will be an uncontrolled stray and some description of its movements, and a better understanding of the source and consequences of the increasing number of dogs micro-chipped outside the UK which become stray.

### Structure of the national population of cats in GB

**Fig 7:**
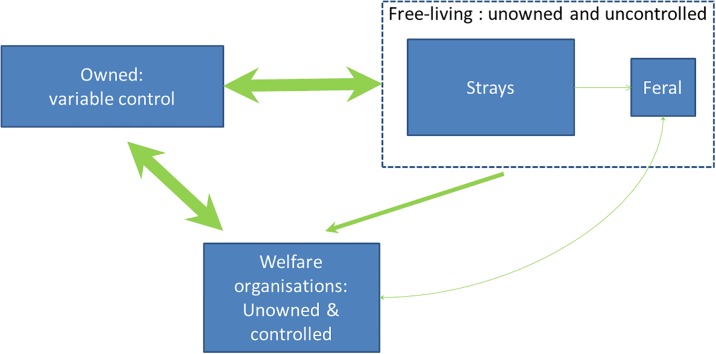
The structure of the British cat population: Sub-populations and their interactions.

Ownership classes of cats are more complex, poorly defined and may not be exclusive (Fig 7). In addition to the class of owned and completely supported pet cats (i.e. confined within homes or free-roaming cats permitted access to them) there appear to be several sub-populations with limited or no control or support and a complicated ownership status (S2 Text). These include cats which are claimed to be owned but appear to receive little veterinary support (Table 2), or alternatively are owned (and may receive both veterinary support and food) but are never permitted into a home and are unlikely to be controlled (Table 2). There are also cats which are not claimed as owned but which may receive some support, e.g. ‘semi-owned’ *sensu* Zito *et al*. (2015); which may include being fed regularly, irregularly or occasionally away from homes, or treated *pro bono* when presented to veterinarians as sick or injured [29]. In addition, there are cats homed in welfare centres, and there may also be small colonies of unsupported feral cats which actively avoid humans (S2 Text). A clear definition of these latter classes and an understanding of their sub-population sizes and the processes which drive individual cats from one to another appear missing.

**Table 2:** Owned cats: Interaction of lifestyle & veterinary support. Interpreted from Murray and Gruffydd-Jones (2012).

		Registration with veterinary practise	
		% registered	% unregistered
Lifestyle	% Confined to home	9.1	0
	% Unconfined	75.7	11.2
	% Excluded from home	1.6	2.4

Our categorisation permits the establishment of a loose hierarchy of ownership classes associated with the likelihood and speed of presentation to a veterinarian when diseased; from owned and confined pets > owned and free roaming > owned and excluded = semi-owned > feral. For the later classes it is possible that cats are only presented to a vet when they are either acutely ill or distressed, or even moribund (for feral animals), permitting an extended period of infectivity before diagnosis and isolation. Some cats in the classes with the least access to care may never see a vet. In the event of an animal health crisis involving cats, the cumulative number of those which are unlikely to be brought under control (i.e. confined for the period of critical threat) or which are less likely to receive veterinary support may be substantial. This includes 1.5 million owned cats and up to 5000 feral cats (Table 3 and S2). Unlike the situation with dogs, where the welfare organisations may have spare capacity to care for an increased population in their long-term care for the duration of a disease crisis (either because the rate of abandonment / ceding goes up or the rate of adoption declines), the assistance the charitable welfare sector might provide to contingency operations involving cats may be small; as there appears to be very little spare capacity to care for cats, and the number of un-owned or semi-owned animals which may benefit by being brought rapidly under control is substantial (S2 Text).

**Table 3:** Quantitative estimates of cat populations and their interactions.

		Source			Working Estimate
		Clark *et al*. (2012)	Stavisky *et al*. (2012)	Murray *et al*. (2015)	
		Survey of welfare organisations (2009)	Survey of welfare organisations (2010)	Survey of households (2011)	Mean estimate [mean % of cats entering care]
Estimated population of owned cats				10,114,764	**10,114,764**
Estimate of annual numbers entering care		131,007	156,826		**143,917**
Standing population in care		14,366 (420 of 748 responses)	18,053 (208 of 536 responses)		**16,209 [11.2%]**
% recycled into the owned population	reunited	2.7	1.4		**2.1%**
	rehomed	85.3	77.1		**81.2%**
% of cats entering care euthanised		5% (5,064/101,257)	13.2% (12,989/98,348)		**[9.1%] representing 13,096 cats**
% euthanized as feral		3%..(56/1849)			**3% representing 392 cats**
% of cats entering care released as feral (TNR)			3.6% (3,512/98,348)		**[3.6%] representing 5,181 cats**

## Discussion

Maps of the density of cats and dogs, as well as estimates of those which may be uncontrolled, have value in a number of fields including policy development and implementation, contingency response, research into the epidemiology of zoonotic and veterinary diseases as well as the impact of cats and dogs on the environment as subsidised meso-predators. Previous work has established some of the associations between pet ownership and demographic classes in GB such as household size [1,20,22,23,30], degree of education / profession [20,22], income [30], rural location [20], owner age or sex profiles [20,22,23] and produced national estimates of pet population size [1,20], but the spatial description of pet ownership has been largely neglected. Here we estimate for the first time a complete national description of the density of owned pet cats and dogs across GB. It is also worth noting here the primary purpose of our maps; to provide an evidential estimate of the number of pets in disease control or surveillance zones (typically of 3km and 10km radius around a focal case) or at larger spatial scales (e.g. within towns, cities or proximal to significant wildlife sites such as national parks).

### The map as a spatial model: Testing the plausibility of its assumptions

As the only quantitative validation we can perform is limited in its value, the plausibility of our maps are must be judged on the quality of our modelling approach, its assumptions, as well as corroboration from qualitative evidence. Three principle assumptions involve the choices made by owners when selecting a veterinary practise (accessibility) and the choices veterinarians make in locating practises (free-market) and FTE as a measure of the distribution of cats and dogs. British owners identified accessibility as their primary criterion when choosing a veterinarian [31] and we relax the strict requirement of this assumption by including a limited spatial smoothing of FTE to account for owners close to catchment boundaries using other nearby practises. This assumption remains valid as long as any variation in the proportion of people using more distant vets does not have a strong spatial component. Conversely, Murray and others also examined distance to vet as a potential explanatory variable in the factors associated with the registration of cats with a practise [30] and neutering [21] and found no relationship using univariate tests, nor did they suggest an association between registration and the number of cats present in the household. We note that this study does suggest that pet ownership (pets.household^-1^) is likely to be higher in homes that are also more distant from a practise (i.e. are in large rural catchments) and that the interaction between these two factors may be important. Our free-market assumption is corroborated by the obvious bias veterinarians have in siting practises in more densely populated areas (Fig 1) and is more objectively supported by examination of the variation in modelled veterinary activity across a fine scale map of residential addresses (FTE.household^-1^; not shown), which is surprisingly small and spatially consistent, suggesting that veterinary activity has indeed distributed itself according to free-market principles. Finally, as most dogs (77%; [25]) and an even greater proportion of cats (86.4%; [21,30]) are registered with a veterinary practise, and veterinarians in small animal practise treat mainly cats and dogs (95.5% combined; [32]) we believe the distribution of small animal veterinarians does make a worthwhile proxy for the distribution of cats and dogs.

The maps shown here are also subject to a series of implicit ‘null’ assumptions regarding the spatial variation of a number of factors i.e. we assume no large-scale variation in their effect on FTE across GB. Thus we assume that there is no underlying compositional variation in the activity of practises (e.g. rural FTE = urban FTE, or southern FTE = eastern FTE); that there is no substantial variation in the species mix of client lists, either with respect to the proportion of cats and dogs, or of other small animal species (e.g. FTE is a compositional factor, a reduction in the proportion of consultations involving one species requires an increase in another). Finally we assume that there is no spatial variation in the value owners place on pets; which combines a number of potential owner behaviours, including a variation in their likelihood of registering with a vet, and variation in the species specific rate of veterinary consultations per pet (e.g. owners in some areas returning with the same pet more frequently). Including such factors as part of the compositional variation in FTE would be simple if robust spatially explicit quantitative evidence were available; unfortunately they are not. We spent some time debating how apparently intuitive factors might be included on the basis of sparse evidence e.g. spatially independent variation in the densities of cats and dogs implied by Downes *et al*. (2011); but often could not even agree a direction of effect, let alone its size. We discuss later the potential of using fine-scale maps of demographic / socio-economic character to inform either the direction or scale of such additional spatial variation in FTE.

It is important to contrast the scale of our mapping (temporal and spatial) and those scales at which we require the model to be informative. FTE essentially represents consultations at a practise across a year and we note practises usually host more than one veterinarian. The smallest-scale for which we require the model to be useful (a disease control zone of 28.3 km^2^) is likely to include many practises. In both contexts substantial variation in the daily experience of an individual veterinarian is expected, but the combination of factors affecting FTE will tend towards the mean national experience with increasing scale. Thus we are most concerned with systematic large-scale unidirectional variation in factors affecting FTE which have the potential to reduce the utility of our maps. Currently none are apparent, though some may be subsequently informed by the recent creation of a number of large-scale studies of veterinary practice e.g. [32].

### The map as a spatial model: Qualitative validation

The plausibility of our maps can also be evaluated against the limited empirical evidence available. Firstly, our description of veterinary activity seems realistic, with 1 FTE representing a mean of approximately 10 consultations.day^-1^ (assuming 200 clinic days per year and a client list including mainly cats and dogs undergoing annual checks).

Secondly, we were concerned that the descriptions of some of our catchments indicated that they may host too many pets (i.e. 11,558 dogs). Asher *et al*. (2011) surveyed all veterinary businesses across GB and enquired about the number of dogs registered at each, which ranged from 100 to 20,356 (from a total of 103 responses: 3.7% of practises questioned: mean of 1.32 practises per response) which suggests the largest practises serve approximately 15,400 dogs. Alternatively, although we only estimate a population of 2,187 dogs in our most populous catchment (43,646 homes), applying previously published *per capita* rates of dog ownership suggests it should support a register of either 14,451 or 11,807 dogs. The former calculation includes the mean number of dogs. English household^-1^ of 0.43 [1], and the proportion registered with a vet as 0.77 [25]. The second calculation follows Asher *et al*. (2011) completely and used the mean proportion of homes with a dog (23.9%), and the mean number of dogs in a dog owning household (1.47) as well as the proportion registered with a vet. As well as suggesting that our catchments are not inappropriately large, we note that difference between our estimate of dog population, based on the provision of veterinary services (2,187), and one calculated simply using *per capita* rates (at least 11,800), is substantial; indicating the requirement of a spatially sensitive proxy for pet density in useful models.

Our estimates of cat density are similar to those reported elsewhere. Churcher and Lawton (1987) conducting a door to door survey across a village in a rural context during 1981 classed densities into four ranges [33]; their most frequent class described densities between 1 and 4 cats.ha^-1^ (100–400 cats.km^-2^), though densities of up to 1100 cats.km^-2^ were found in some locales. A study in suburban Bristol during 2002 estimated 19% of the households in the area owned cats with an mean of 1.43 cats.owner^-1^ producing 229 cats km^-2^ and 0.27cats.household^-1^ [34], whilst densities between 132–1579 cats.km^-2^ [19] and between 230–1012 cats.km^-2^ [24] were estimated more recently by extrapolation from surveys of householders in mainly residential urban and suburban areas. All of these densities are considered common in our model (Fig 4) and as none of the empirical estimates included areas where we generally find the extreme densities of cats (e.g. metropolitan city centres) we believe that our estimates for areas of density of < 2500 cats.km^-2^ are reasonable, based on the high densities of both homes and veterinary practises in some urban areas. Although we have suggested that the few distinct small rural sites with densities of cats > 2500 cats.km^-2^ are unrealistic, we have no direct evidence to support this. Such densities might be considered plausible when considering the high mean rates of cat ownership found in some studies (e.g. 0.23, [1]; 0.33, [23]), and the mean number of cats per cat owning household found in some locales (e.g. 1.91 cats, [24]). Combining these maximal rates would only require densities of 4000 households.km^-2^ to achieve our threshold; a condition easily exceeded in many housing developments in GB. In addition, considering the spatial variation in cat ownership suggested in this study and elsewhere [24], and the demographic / socio-economic factors associated with an increased likelihood of cat ownership (e.g. rural location, improved education, higher income [20]) and trends for these factors to be co-located in small affluent country towns or villages, we feel that our arbitrary threshold for an unrealistic density of cats is conservative and might be exceeded at small scales in some places.

Our maps illustrate in detail the broad themes often described in other studies. Thus the density of cats correlates with the density of humans resulting in the high densities of both cats and dogs in urban areas [19,23,24]. Although our description of the number of pets per household is not directly comparable with the rate of ownership (as it is confounded with the number of pets per owner) the substantial and almost universal increases it shows across most rural areas is corroborated by previous studies also showing increased rates of ownership in rural areas of both dogs and cats [20,23,35]. Conversely, Sims *et al*. [19] found no relationship between housing density and the proportion of households that own cats, though we suggest that this may reflect the limited range of locations and contexts which they studied. For dogs there appears to be very little consistently sampled qualitative or quantitative information with which to attempt a validation. Asher *et al*. (2011) described some geographical variation (across 17 randomly selected areas) in the proportion of households with dogs (range: 10–67%) and the number of dogs per owner (range of mean dogs.owner^-1^: 1–2.5) and any product of these estimates (our estimate of dogs.household^-1^) fall within the range we describe; unfortunately their sample sizes are small and their geographic descriptor (areas covered by printed telephone directories) cannot be mapped and so quantitative validation against this study is impossible.

Our general model appears to have failed in a very small number of discrete locations, and predicted unrealistically high densities or rates of ownership. These appear associated with three types of veterinary establishments; large referral centres in cities, veterinary schools which run surgeries, or very small rural catchments served by a large practise (an unusual combination as many rural practises treating pets are small and most rural catchments are large). All of these practises will have registered undertaking activity with cats and dogs, though for each the number of surgeons employed and the number of pets treated did not appear to fit our free-market assumption. For some referral centres, although they may mainly treat pet cats and dogs, the generally specialised nature of their services, along with their indirect relationship with owners suggests that they service much larger areas across their principle client base, neighbourhood veterinary practises delivering primary care. Unfortunately the diverse nature of the veterinary profession in GB, including large independent referral centres with varying degrees of specialisation and varying provision of walk-in services, some large mixed practises hosting referral services for small independent veterinarians, as well as some small practises running specialised veterinary referrals alongside their general practice, means that current data cannot identify the scale of referral activity and permit this to be mapped independently at a larger scale. A few teaching centres and the very few rural practises generating high pet densities occurred because the relatively large number of FTEs at those locations were there for reasons other than treating pets i.e. for teaching, or where pets are treated but as a minority activity at large mixed practises mostly undertaking equine or farm animal work. In both of these cases we cannot identify the proportion of the FTE allocated to the care of pets and assume they operated as neighbourhood practises, causing an overestimate of their activity and the proportion of the pet population served within their catchments. The small number of homes in some of these aberrant rural locations amplifies the excessive prediction of pet populations into unrealistic predictions of the rate of ownership.

### The map as a spatial model: Quantitative validation

The quantitative validation undertaken here is the best available, though may be problematic as it relies upon two small historical studies of cats, both achieved through survey and extrapolation [19,24]. Firstly, the interval between the collection of data for the most powerful study by Sims *et al*. [19] and the acquisition of the data describing the activity of practises here is 10 years, long-enough to see considerable changes in the local densities of both humans and cats as well as socio-economic changes affecting the distribution and activity of veterinary practices. Even in the 15 months between interrogations of the RCVS register the dynamism of veterinary activity across GB was notable, and we suggest that 10 years is too long an interval for robust validation. Secondly, both sets of validation data were limited in size and scope, with the study by Thomas *et al*. [24] only representing nine independent catchments with its 16 locations. Together the validation data represented only 0.01% of catchments modelled here (39 of 3918) or 0.0002% of 1 km grid squares across GB (46 of 232,835) and both empirical studies were undertaken in the same restricted contexts (i.e. mainly residential urban and suburban land-uses). Further, as expected, the smallest and most restricted study observed the smallest range of densities [24] which suggest that both studies may poorly describe the range of local cat densities at the national scale. We suggest that robust validation would require a large survey across a national scope to capture a suitable range of spatial and demographic contexts. Thirdly, both empirical studies calculated their densities by extrapolation, and this may have produced a substantial error in their estimates. In part we have anticipated this using the intra-class correlation coefficient for validation which does not assume that either estimate at a location (empirical or modelled) is true, and the generally symmetrical variation of empirical estimates around the model output may either represent the genuine small-scale variation in cat density between survey locations (scaled up to represent 1km^2^) or the amplification of un-biased error through extrapolation around a true value approximate to those we estimated. Finally we note the inadequacy of using legacy spatial structures (e.g. 1 km grids aligned to the British National Grid) to describe and compare estimates generated at other scales and urge future studies to completely and accurately describe their study areas (location, area, extent; ideally as vector polygons) to permit robust comparison with other work.

Our quantitative validation may indicate where and why the inclusion of demographic / socio-economic factors into the modelling process may be beneficial. Four locations used for quantitative validation describe much higher densities of cats than we predict ([19], Fig 5); all are in densely populated urban locations, though with a relatively limited availability of veterinary services. This difference may be real and could arise from a much lower proportion of pets from these neighbourhoods being registered with a practise for either cultural or socio-economic reasons. If true, the inclusion of additional predictive factors to refine the description of pet density and ownership has particular value in areas such as these. Omission of these four exceptional validation points substantially improves the correlation used for the quantitative validation (agreement of 0.67 and consistency of 0.69) and may better describe the fit of the model across GB given the relative rarity of similarly dense housing in city centres (Fig 2).

### Structures of the pet population in GB: Components and interactions

Our national-scale overview of the dynamics of cat and dog ownership was facilitated by the publication of a series of contemporaneous descriptions of key component sub-populations (e.g. character, size) and their interactions [1,26–28] which we have simply interrelated and cohered to a common scale. In broad terms it permits the contextualisation of the study of individual component sub-populations, though we also use it to infer the character and size of difficult to study and poorly reported sub-populations such as feral cats. It also permits an appreciation of the numbers of pets which might not be controlled during a crisis, or which may not have access to a vet to permit the timely diagnosis of disease. In addition, it also identifies the most important gaps in our understanding of the complete cat or dog systems, essential for the robust predictive modelling of the epidemiology of diseases of policy or veterinary concern. A number of striking features have become evident.

The sizes of the owned sub-populations of both species dwarf other ownership classes. For dogs this may well mean that during a period of disease crisis, all animals are effectively under control, and even the 23% of dogs currently unregistered with a veterinary practise [25] could be promptly presented to a veterinarian if necessary. Conversely, for cats, despite their greater rate of registration with a practise (86.6%) [21,30], substantial sub-populations may never be brought under control, health checked or vaccinated, even if the majority of such cats are claimed to be owned. We also note the paradoxical observation that despite similar national population sizes for cats and dogs [1] and a greater proportion of owned cats being registered with a veterinary surgeon, cats are only presented to vets at approximately half the rate of dogs [32]. Absent or limited veterinary support is taken here as a proxy for the risk of a disease of policy concern being identified late in its epidemiological development because animals may only be presented to a vet when acutely ill (or even moribund, as is likely with feral cats) and well after the onset of infectivity. This may produce a prolonged period of transmission to other uncontrolled cats and secondary hosts (including wildlife and man), which may prejudice both disease surveillance and control. More complicated yet are the diffuse definitions of ownership classes for cats, based as some are on an owner’s self-declaration and their perceptions of their responsibilities, alongside a cat’s apparent free-choice to move freely between sub-populations (feral ↔ unowned but supported ↔ adopted into a home). Other authors have described the particular difficulties in studying the structure, size and dynamics of the un-owned populations of cats and the difficulties in estimating their dynamics [27] though it is specifically these sub-populations which are usually considered of most concern because of their potential to damage the environment, harbour both endemic and exotic diseases as well as their potential role to amplify the risk of wildlife zoonoses being vectored into the human population. However, our analysis suggests that intervention (e.g. public information, education) might be most cost effective when targeted at the significant numbers of self-declared or *de facto* owners of cats who perceive little value in veterinary care, rather than field operations against small colonies of feral cats.

It is evident that the flux of pets between the standing owned and unowned populations (i.e. those housed for the long term by welfare organisations) is over 10-fold larger than the standing population in care (Tables 1 and 3). Should a scenario occur where members of the public no longer wish to acquire pets from welfare organisations, or alternatively pets were abandoned or ceded at an increased rate, there is the potential for pets to accumulate rapidly in long-term care. For dogs, there may be a short period before this becomes problematic, as the sector caring for dogs may have spare capacity [26,27] and micro-chipping will allow organisations to reunite dogs with their owners despite owner passivity or negligence. Unfortunately, the welfare organisations caring for cats operate much closer to their capacity [26] and they are likely to rapidly reach a point where no more can be accepted during a period of crisis.

### Map as a tool

One of the primary purposes of this study is a tool to permit the rapid estimation of the number of pets falling within statutory protection and surveillance zones following a focal case of notifiable disease (minimum radius of 3 km and 10 km respectively). Uniquely, this study permits this from any point across GB. Whilst the accuracy of the estimation is unknown at small spatial scales (e.g. postcode units in cities), we believe it provides an unbiased estimate at individual 1 km^2^, and the quantitative validation suggests that this approach should be robust when considering areas at least as large as 28 km^2^ and 314 km^2^ (i.e. estimates tend toward the mean). Where we identified potentially unrealistic densities of pets or rates of pet ownership we note that these are both very few and very limited in extent; as they will only lead to slightly increased estimates in the number of pets when aggregated into larger scales, this conservative over-estimate is unlikely to be operationally problematic.

A number of reasons have prompted us to not combine the two independent tools presented here, estimates of density of pet populations and estimates of the uncontrolled proportion of those populations, into a single object. Firstly, space here precludes the necessary validation and discussion of what would be a new map. Secondly, the combination would require some form of independent spatially explicit validation, and as far as we are aware there is no data to support this. Thirdly, as with any spatially explicit data, there are complicated interactions between the variability and uncertainty of the data at its natural scale, at its scale of study and at its scale of representation. These impinge directly on value judgements associated with an applied inference; which vary between academic, operational, theoretical or real decision-making scenarios. For example, if we are correct in assuming a national population of feral cats to be as high as 5000 animals, workers would need to make assumptions as to how this is represented at smaller scales and how this might influence their final inference. Thus zoonotic or veterinary disease risk assessment, which might wish to combine our tools, would typically integrate this with additional quantitative factors pertinent to their specific disease scenario and at a scale meaningful to their purpose. A generic combination at an arbitrary scale here is thus less useful.

## Supporting information

S1 Text Construction of the spatial model describing the density of cats and dogs across Great Britain. (DOCX)

S2 Text Review: Size, character and interactions of key sub-populations of the British populations of cats and dogs. (DOCX)
