# Supplementary material for: A first estimate of the structure and density of the populations of pet cats and dogs across Great Britain
Source: PLoS One. 2017 Apr 12;12(4):e0174709. doi: 10.1371/journal.pone.0174709 (PMC5389805; doi:10.1371/journal.pone.0174709)
Supplement: S1 Text — (DOCX) [file pone.0174709.s001.docx]

# S1

# Construction of the spatial model describing the density of cats and dogs across Great Britain

## Overview

Registered veterinarians are likely to treat most owned cats and dogs in Great Britain (GB)(86.4% of cats [1]; 77% of dogs [2]), and these two pet species make up the majority of veterinary consultations for small animals (95.5% [3]). As veterinarians operate in a free-market in GB, the distribution of their activity should reflect the density of their principle clients; the owners of cats and dogs. Locations of practises likely to treat pets (e.g. excluding specialist practices for large animals, equine services, zoos as well as those practices requesting anonymity) were obtained from the authoritative register of prescribing and dispensing practises maintained by the Royal College of Veterinary Surgeons (RCVS) as a full postcode, along with a count of the number of veterinarians registered at each. The location permitted the calculation of exclusive catchments around each practise based on client travel time (isochrones) and the association of every residential postcode unit (and by extension residence) with a veterinary practise.

At the national scale, estimates of the number of cat and dogs, as well an accurate description of the number of prescribing veterinarians and their practises permits us to generalise about the number of cats and dogs seen by vets at every practise. We extend this to smaller spatial scales by recognising that (1) practise catchments may differ in their area and the number of households they serve, and (2) that the activity of a practise in treating cats and dogs is likely to vary dependent on the number of veterinarians they employ. Here we combine both factors to estimate the first map of the density of pet cats and dogs across GB. We recognise that improvements to the methodology are potentially possible, for example adding demographic / socio-economic factors to adjust the likelihood of cat or dog ownership (e.g. [4]), as well as robust quantitative information on the spatial variation of the behaviour of veterinarians and their practises (i.e. activity and composition of client pets). In addition, future models and validation could be improved by the availability of any spatially explicit empirical estimate of the density of dogs, as well as more robust and general estimates of the density of cats. However, as suitable data to support these enhancements are currently unavailable, we present this study as the best current estimate of the densities of cats and dogs across GB and a first step towards providing superior estimates in the future.

## Calculating catchments

Practises were located on a comprehensive description of the road network across all of GB using OS Codepoint (Ordnance Survey: Southampton) in ArcGIS 10.2 (ESRI: Redlands, California). The road network was constructed from elements derived from OS MasterMap (Integrated Transport Network layer, ITN; Ordnance Survey: Southampton), where each element was given an appropriate transit speed (Table A). Most network features permitted access to practice locations and the calculation of customer access (i.e. catchments). However motorway features simulated their real-world behaviour and could only be reached indirectly across the road network and did not contribute directly to constructing catchment boundaries.

Unique, topologically exclusive and contiguous catchments around each practise location were calculated by computing isochrones between every neighbouring practise across the country (using the ‘Service area’ tool within the Network Analyst extension of ArcGIS), though these were not required to be continuous. Thus a practise close to one motorway junction might serve clients clustered around a successive junction when intermediate addresses might be served by alternative practises. In some towns and cities more than one practise might be located within the same postcode unit, in which case the FTEs for those practises were combined to represent a single catchment. In areas with very high densities of practises, exclusive catchments could become so small (only a few 100’s m across) that these were also combined to more realistically represent the locale (again preserving the sum of their veterinary activity). Similarly, in some areas the structure of the road network resulted in modelling artefacts, which whilst logically true complicated subsequent GIS operations and calculations, and these too were combined. It was not computationally possible to calculate catchments for all practises across the complete travel network in one pass; therefore the task was achieved by computing overlapping local groups of approximately 50 practises and merging these.

**Table A. Network features and their transit speeds**

| Network Feature | | Speed (kph) |
| --- | --- | --- |
| Motorway | Dual carriageway | 110 |
|  | Single carriageway | 90 |
|  | Slip road | 70 |
|  | Other features | 50 |
| A roads | Dual carriageway | 90 |
|  | Single carriageway | 70 |
|  | Slip road | 50 |
|  | Other features | 30 |
| B roads | Dual carriageway | 70 |
|  | Single carriageway | 50 |
|  | Slip road | 40 |
|  | Other features | 30 |
| Minor roads | Dual carriageway | 50 |
|  | Single carriageway | 40 |
|  | Slip road | 40 |
|  | Other features | 20 |
| Local streets | Dual carriageway | 50 |
|  | Single carriageway | 50 |
|  | Slip road | 40 |
|  | Other features | 20 |
| Private roads | Dual carriageway | 50 |
|  | Single carriageway | 50 |
|  | Slip road | 40 |
|  | Other features | 20 |
| Pedestrian streets |  | 10 |
| Alleys |  | 10 |

## Constructing the final map

Whilst the description of the network properties of catchments were always consistent, regardless of the number of times one was calculated in differing local groups, the only algorithm available used to describe the space between network edges could generate small variations in catchment boundaries when run multiple times, especially where the travel network was sparse or the catchment was at the edge of a local group. These variations produced anomalies (small gaps and overlaps) when local groups were combined into a single mosaic where catchments were required to be unique and exclusive and their mosaic topologically continuous. Small gap or overlap anomalies in catchment boundaries (i.e. within 10m) were resolved using an unsupervised approach (Integrate>Data Management: ArcGIS), whilst larger overlap anomalies were resolved individually by identifying the most appropriate catchment boundary and/or using the edge-matching tools available within ArcGIS; the anomalous boundary was usually obvious when visualised along with the travel network. Larger gap or edge anomalies were addressed by intersecting catchments with a fine-scale map of postcode units (OS Codepoint) and using the geographical description of units exclusive to each catchment (defined by the location of unit centroids) to define the boundary of each catchment; this was particularly useful in coastal areas where the road network did not extend close to the high-water mark. Finally, the national mosaic of catchments was clipped to a detailed map of the GB coastline (OS Boundary line; Ordnance Survey: Southampton). Two attributes were calculated for each catchment; its area (km^2^) and the number of households, derived from the sum of the residential delivery points described in every postcode unit exclusively associated with each catchment.

The modelling approach (exclusive catchments across a travel network) assumes that customers only select their most accessible practise (closest in travel time) which produces two principal concerns. Firstly, a number of factors may lead a minority of customers to choose a neighbouring though more distant practise and some method of representing this is seen as desirable. This seems most likely for clients living close to catchment boundaries. Secondly, exclusive catchments can occasionally produce an unrealistically discontinuous representation of veterinary services because of the large variation in the activity of neighbouring practises; in turn this produces substantial variation in the estimates of pet density and the rate of pet ownership at small geographical scales. To address both issues we smooth the distribution of veterinary FTEs at the scale of the postcode unit; achieved using an exponential Kernel interpolation model (ArcGIS; ESRI) with a bandwidth of 2 km and replacing any impossible smoothed values (i.e. < 0 FTEs household^-1^) with the lowest positive value (2.3 x 10^-5^) and rescaling the smoothed values to ensure the national FTE of vets remains constant. In practise this cap on the lowest values was only applied to 0.02% of post codes and the rescaling factor was 0.9968 (i.e. minimal rescaling) indicating that the smoothing achieved its purpose of spreading veterinary effort across the edges of large catchments without bias. Two visualisations of the map were produced; a direct summation of the numbers of cats and dogs across a conventional 1 km raster (the area of postcode units represented proportionally in each grid cell) and one aggregated to postcode district for the description of pets.household^-1^.

### Practise activity

No empirical data were available to determine the nature of the relationship between the number of veterinarians registered at a practise (*v*) and the activity of the practise specifically supporting cats and dogs expressed as full-time-equivalents (FTEs). Thus we simply assumed that sole practitioners usually worked at 1FTE; that a practise with 10 vets approximated 6 FTEs; and that one using the services of 20 vets approximated 10 FTEs and fitted a quadratic relationship:

FTE = -0.015*v*^2^ + 0.8*v* + 0.215

Very large veterinary centres (mainly referral centres and clinics associated with teaching schools) with more than 20 registered vets undertaking small animal practise were capped at 10.88 FTEs. This assumption expresses our belief that large practises tend to employ more part-time staff as well as offering a broader range of services requiring the use of practitioners specialising in unusual species (i.e. not cats or dogs) or specific techniques which may permit more complex diagnosis and treatment but would not necessarily result in more pets being treated per registered vet. We note that some practises in our data appear to treat cats exclusively, though they are few and generally operate as sole practitioners, and we have ignored their species specificity for this first national-scale exercise.

## References

1. Murray JK, Gruffydd-Jones TJ. Proportion of pet cats registered with a veterinary practice and factors influencing registration in the UK. Veterinary Journal. 2012;192(3):461-6. doi: 10.1016/j.tvjl.2011.08.035. PubMed PMID: WOS:000306195700040.

2. Asher L, Buckland EL, Phylactopoulos CI, Whiting MC, Abeyesinghe SM, Wathes CM. Estimation of the number and demographics of companion dogs in the UK. Bmc Veterinary Research2011.

3. Sánchez-Vizcaíno F, Jones PH, Menacere T, Heayns B, Wardeh M, Newman J, et al. Small animal disease surveillance. Veterinary Record. 2015;177(23):591-4. doi: 10.1136/vr.h6174.

4. Murray JK, Browne WJ, Roberts MA, Whitmarsh A, Gruffydd-Jones TJ. Number and ownership profiles of cats and dogs in the UK. Veterinary Record. 2010;166(6):163-8. doi: 10.1136/vr.b4712. PubMed PMID: WOS:000274490600012.
