# Supplementary material for: A first estimate of the structure and density of the populations of pet cats and dogs across Great Britain
Source: PLoS One. 2017 Apr 12;12(4):e0174709. doi: 10.1371/journal.pone.0174709 (PMC5389805; doi:10.1371/journal.pone.0174709)
Supplement: S2 Text — (DOCX) [file pone.0174709.s002.docx]

# **S2**

# Review: Size, character and interactions of key sub-populations of the British populations of cats and dogs

We reviewed the contemporary literature describing the sizes and dynamics of key components of the national populations of cats and dogs, with specific reference to the degree of control exercised over pets and their relationship with a veterinarian. This permitted us to establish a complete and coherent matrix of descriptions to help describe the structure of the national populations, and aggregate empirical the descriptions of each pet sub-population into one of a number of ownership classes for both cats and dogs. We then scaled the sizes of each of the key sub-populations of pets against the estimate of the owned population [1] which relates to data collected in 2011, and attempt to estimate the relative flow of pets between them using contemporary estimates of sub-populations and processes to ensure comparability. Although many of the estimates of sub-population sizes are uncertain (many are based on questionnaire surveys) our intention is to attempt a complete albeit approximate description of the relative sizes of major standing sub-populations, and the major movements of pets between them.

## Dogs

Owned dogs dominate the national population in GB. The standing sub-population of unowned dogs, mainly housed by charitable welfare organisations, represents only 0.002% of the owned sub-population across GB (Main text, Table 1). As most unowned dogs are in care of some sort they are likely to have timely access to a veterinarian, whilst only 77% of owned dogs will be registered with a veterinary practise [2]. However, the substantial sub-population of owned dogs not registered with a practise is nominally under the control of their owners and could be presented to a veterinarian if required.

There is no robust estimate of the standing sub-population of unowned dogs, which is almost completely maintained and controlled by the welfare organisations, though two independent studies report similar numbers of dogs held in long-term care (Main text, Table 1). Unfortunately, neither study estimates the un-owned sub-population across the full scope of the welfare organisations surveyed and both report survey results from a limited set of survey responses. As the entire standing population is likely to be less than twice the reported numbers (as other estimates reported in both studies appropriately scaled to the whole sector rarely double) we conservatively assume a figure of twice the mean value, 21,837; which represents 0.0019% of the owned population. It might be hoped that the recently implemented compulsory identification and registration of dogs will facilitate a more robust quantification of the dog population across its different ownership classes and the processes driving dogs between them. For example, it is not clear how many individual dogs participate in the annual flux between ownership classes (i.e. some dogs may repeatedly stray and be repeatedly reunited or rehomed) and previous authors have identified the standardisation/quality of record keeping in some welfare organisations as a major barrier to a more complete understanding of this issue [3,4].

The instantaneous proportion of dogs entering the temporarily stray and uncontrolled component is very small and probably represents < 3.0 x 10^-5^ % of owned dogs (1 in 30,000); assuming the annual total number of strays represents approximately 0.01% of the owned sub-population (as it did in 2011; Table 1) and a consistent daily rate of straying. Nonetheless, this represents a turnover of 345 strays.day^-1^ which run uncontrolled across GB. There is some evidence that the rate of straying in dogs shows both temporal and spatial variation [5]. Although the absolute temporal variation is substantial (range 97,000 to 126,000 strays.year^-1^ between 2008 and 2014), it has shown no sustained trend and is sufficiently small as a proportion of the owned population to be simplified to a mean total number of 113,000 strays.year^-1^. Insufficient data exist for the spatial variation to be modelled at the fine spatial scales used in this study, though substantial regional differences in the number of dogs straying are likely [5].

Most uncontrolled strays appear to be collected by local authorities who kennel dogs for the statutory minimum of 7 days, and manage to quickly reunite 50% of strays with their owners [5]. A smaller number of stray dogs are independently collected by welfare organisations and these also reunite approximately 10% with their owners (Main text, Table 1). The estimate reported by Stavisky *et al.* (2012), based on questionnaires to welfare organisations, does not permit the distinction between dogs received directly into the care of welfare organisations and those that have been passed to them by local authorities after the statutory period of kennelling. We have assumed that the size of the population they report as ‘found as stray/lost’ by welfare organisations mainly represents dogs passed on by local authorities as it approximates the population stated to have been transferred to these organisations by the authorities in 2010 [5]. The increasing popularity of micro-chipping dogs appears to have substantially increased the proportion of dogs returned to their owners, and the mandatory chipping of dogs required from April 2016 is expected to see further increases in the efficiency of this process. The voluntary ceding of dogs to either local authorities or welfare organisations also occurs though it is not clear how prevalent this is, with local authorities suggesting it is a small proportion of those they collect, whilst others report it represents over 56% of dogs received at welfare organisations [4]. Importantly ceded dogs are always under control. Abandoned strays and ceded dogs nominally enter the standing population of dogs in care, though both local authorities and welfare organisations run re-homing programmes and a substantial proportion are found new homes and return to the sub-population of owned pets (Main text, Table 1).

## Cats

Using a similar approach to that taken for dogs, we identify a number of exclusive sub-populations of cats and estimate their sizes scaled to the size of the owned sub-population. Murray and Gruffydd-Jones (2012) describe the structure of the owned population of cats in GB (Main text, Table 2), including the substantial proportion confined entirely within the home (9.1% or 0.92 million) which appear uniquely protected from contagious diseases of policy concern. A lower proportion of cats (3%) were reported as completely confined by Sims *et al.* (2008), though this was taken from a small survey in one location and we have used the more robust and general estimate for our calculation here; though this difference may indicate the potential for spatial variation in this type of cat ownership driven by cultural and socio-economic factors (e.g. high-rise apartment living). The majority of owned cats (8.8 million) are usually permitted to roam free, though could presumably be confined during a period of crisis. However, this substantial sub-population of cats is uniquely placed to vector zoonotic infection from any number of sources directly into intimate contact with humans. Of these over 1.1 million are unlikely to be registered with a veterinary practise indicating that they may only present for diagnosis late in their pathology (Main text, Table 2). Of most interest however are the 0.4 million cats which are said to be owned but live entirely outside homes (Main text, Table 2); it is also significantly less likely that these cats will be registered with a veterinary practise [6]. As well as the potential difficulty in securing these cats during a crisis we also anticipate that most will not be house-trained and suggest that those who claim ownership will be reluctant to confine them.

Despite the absence of a publicly funded service to manage stray cats the estimate of the number of cats entering the care of welfare organisations is larger than for dogs (Main text, Table 3), with a smaller proportion being ceded by owners [4], and a much smaller proportion found stray returned to their owners (Main text, Table 3). Together this suggests two potential scenarios of interest here; a large self-sustaining sub-population of free-living cats, or the increasing commoditisation of cats. The presence of a large sub-population of free-living unowned cats reproducing without constraint might produce the patterns of acquisition reported by welfare centres, which helps explain the relatively high submission rate of apparently homeless cats to welfare organisations as sick, lost or stray; supplementing the owned cats which have been ceded, abandoned or lost which are also acquired. As many owned cats are not marked it is difficult to determine the source sub-populations for cats received by welfare centres, though we attempt to sketch the probable sizes of their sub-populations below and suggest that a large number of free living unowned cats is unlikely. Alternatively it may reflect a much lower financial and emotional investment by owners in their cats, compared to their dogs, which results in a generally small effort to recover lost or straying cats, a readiness to eject problematic animals and simply replace a lost cat with another from a welfare centre. This possibility is supported by the paradoxical observation that the proportion of veterinary consultations undertaken for cats is substantially lower than that for dogs (25.6% vs 69.9% for cats and dogs respectively [8]) despite similar numbers of owned cats and dogs in GB [1] and with cats presenting a higher rate of registration with veterinarians [2][6]. If the commodification of cats is occurring then this may also be reflected in owner’s willingness to seek veterinary care or assist the authorities acting against a disease of policy concern by confining or controlling their pet.

Estimating the size of the free-living unowned sub-populations of cats (either semi-owned or truly feral) has been identified by many as difficult. Here we note that two studies quantify some aspects of the apparently free-living population where they occur within welfare organisations and we combine estimates from a number of studies (Main text, Table 3) to suggest up to 392 cats.year^-1^ are euthanased as ‘feral’, presumably because their behaviour caused problems in captivity. As the behaviour of cats around humans may be the only distinctive difference between the two unowned sub-populations (with semi-owned cats being comfortable around humans whilst true feral cats are not) this may help identify the scale of the truly feral population. Alternatively we estimate the number of cats released by organisations operating trap-neuter-release schemes for ‘feral’ cats at up to 5000 cats (Main text, Table 3), although it is not clear if these schemes are restricted to true ferals or includes semi-owned animals. Thus we suggest a national population of feral cats of between approximately 400 and 5000 in 2010 – a very small proportion of the owned population. Very little direct quantitative evidence exists to corroborate these extrapolated estimates. Askew (pers comm.) undertook a field survey of feral cats in 2000 replicating one undertaken in 1986. This covered three areas of England totalling over 400 km^2^ and found a total of 43 colonies, most of which were associated with factories, trading estates and heavy industrial complexes. Most colonies (65%) were in the smallest size class (3-5 cats) though two colonies numbered more than 16 cats. We have assumed this represents a minimum population of 294 cats indicating a decline from the initial survey (assumed to have counted 483 cats using identical assumptions). It is impossible to extrapolate this across a national scale and we note that cats were mostly associated with land-uses which have changed dramatically since both surveys were undertaken (i.e. declined). Again this suggests that true feral cats are very rare across GB and that their colonies are small and thus unlikely to be self-sustaining in the absence of immigration by un-neutered cats from the owned or semi-owned sub-populations.

The size of the stray owned or semi-owned cat sub-population is also difficult to estimate. Here we calculate the annual total estimated to have been rehomed as 116,861 (Main text, Table 3). As 18.6% of owned cats were acquired as strays [6] and 15.3% from rescue centres, we estimate that 142,066 cats.year^-1^ were acquired as strays. This equates to a daily turnover of approximately 390 cats. Comparing the daily turn-over of stray cats with stray dogs, and the broad similarities between the sizes of their owned populations and the annual number received into care (Main text, Table 1 & Table 3), suggests that the size of the standing sub-population of unowned free-roaming cats is also similar to that of unowned free-roaming dogs i.e. negligible. We caveat this qualitative assessment by noting the cultural sensitivities to the presence of free-roaming dogs in GB and that they are likely to be quickly reported and rapidly collected, ensuring no real uncontrolled population. Conversely, uncontrolled cats are ubiquitous, and very little attention is directed towards healthy animals, which along with the absence of publicly funded cat collection might produce a substantial unremarked free-living semi-owned population with individuals drifting between different states of non-ownership as circumstances or their behaviour dictate. However, if this were the case, rates of un-neutered kittens/young cats admitted to welfare organisations [9] would be much higher than that found in owned cats [10], which it is not, suggesting that the sub-population of free-living cats is very small in proportion to the total population.

## References

1. Murray JK, Gruffydd-Jones TJ, Roberts MA, Browne WJ (2015) Assessing changes in the UK pet cat and dog populations: numbers and household ownership. Veterinary Record.

2. Asher L, Buckland EL, Phylactopoulos CI, Whiting MC, Abeyesinghe SM, et al. (2011) Estimation of the number and demographics of companion dogs in the UK. Bmc Veterinary Research.

3. Clark CC, Gruffydd-Jones T, Murray JK (2012) Number of cats and dogs in UK welfare organisations. Veterinary Record 170: 493.

4. Stavisky J, Brennan ML, Downes M, Dean R (2012) Demographics and economic burden of un-owned cats and dogs in the UK: results of a 2010 census. Bmc Veterinary Research 8.

5. Anon. (2014) Stray Dogs Survey 2014. Dogs Trust.

6. Murray JK, Gruffydd-Jones TJ (2012) Proportion of pet cats registered with a veterinary practice and factors influencing registration in the UK. Veterinary Journal 192: 461-466.

7. Sims V, Evans KL, Newson SE, Tratalos JA, Gaston KJ (2008) Avian assemblage structure and domestic cat densities in urban environments. Diversity and Distributions 14: 387-399.

8. Sánchez-Vizcaíno F, Jones PH, Menacere T, Heayns B, Wardeh M, et al. (2015) Small animal disease surveillance. Veterinary Record 177: 591-594.

9. Murray JK, Skillings E, Gruffydd-Jones TJ (2008) A study of risk factors for cat mortality in adoption centres of a UK cat charity. Journal of Feline Medicine and Surgery 10: 338-345.

10. Murray JK, Roberts MA, Whitmarsh A, Gruffydd-Jones TJ (2009) Survey of the characteristics of cats owned by households in the UK and factors affecting their neutered status. Veterinary Record 164: 137-141.
